# Tsw – A case study on structure-function puzzles in plant NLRs with unusually large LRR domains

**DOI:** 10.3389/fpls.2022.983693

**Published:** 2022-10-07

**Authors:** Irene Louise van Grinsven, Eliza C. Martin, Andrei-José Petrescu, Richard Kormelink

**Affiliations:** ^1^ Laboratory of Virology, Department of Plant Sciences, Wageningen University, Wageningen, Netherlands; ^2^ Department of Bioinformatics and Structural Biochemistry, Institute of Biochemistry of the Romanian Academy, Bucharest, Romania

**Keywords:** Tsw, plant NLR protein structure, LRR domain, R protein, immunity

## Abstract

Plant disease immunity heavily depends on the recognition of plant pathogens and the subsequent activation of downstream immune pathways. Nod-like receptors are often crucial in this process. *Tsw*, a Nod-like resistance gene from *Capsicum chinense* conferring resistance against Tomato spotted wilt virus (TSWV), belongs to the small group of Nod-like receptors with unusually large LRR domains. While typical protein domain dimensions rarely exceed 500 amino acids due to stability constraints, the LRR of these unusual NLRs range from 1,000 to 3,400 amino acids and contain over 30 LRR repeats. The presence of such a multitude of repeats in one protein is also difficult to explain considering protein functionality. Interactions between the LRR and the other NLR domains (CC, TIR, NBS) take place within the first 10 LRR repeats, leaving the function of largest part of the LRR structure unexplained. Herein we discuss the structural modeling limits and various aspects of the structure-function relation conundrums of large LRRs focusing on *Tsw*, and raise questions regarding its recognition of its effector NSs and the possible inhibition on other domains as seen in other NLRs.

## Introduction

The basics of a properly working immune system in all organisms is the ability to distinguish self and non-self molecules, after which harmful intruders are forcefully removed while their own cellular integrity remains intact. Conserved microbial patterns, so-called microbe/pathogen associated molecular patterns (MAMPs, PAMPs), are recognized by pattern recognition receptors (PRRs) on the cell surface. However, effectors secreted by pathogens can suppress this pathway and lead to an infection. Plants in turn have acquired cytoplasmic resistance (*R*) genes, or nucleotide-binding oligomerization domain (NOD)-like receptors (NLRs), that can detect effectors and trigger an immune response (Jones and Dangl, 2006). NLRs are a diverse group of proteins. Most NLRs contain a variable N-terminal domain, as well as a nucleotide-binding domain (NBD), and a C-terminal leucine rich repeat (LRR) domain. The different N-terminal domains are used to classify NLRs into three different groups: the coiled-coil (CC) NLRs (CNLs), the Toll-interleukin receptor (TIR) NLRs (TNLs), and the Resistance to Powdery Mildew 8-like domain (RPW8)) NLRs (RNLs). The remainder, comprised of NLRs without an N-terminal domain, are classified as NBS-LRR, or NL, receptors (Duxbury et al., 2021).

The central NBD of NLRs contains a NB-ARC, a nucleotide binding (NB) adaptor shared by human Apaf1, plant resistance proteins, and nematode CED-4 (ARC) (van der Biezen and Jones, 1998). The NB-ARC domain is a molecular activation switch that can bind adenosine 5′-diphosphate (ADP) and adenosine 5′-triphosphate (ATP) (van Ooijen et al., 2008; Takken and Tameling, 2009; Maekawa et al., 2011; Williams et al., 2011). In the absence of the pathogen effector, the LRR of an archetype NLR forms a tight horseshoe shape in which the N-terminal domain and the NB-ARC domain are nestled (Kobe and Kajava, 2001). The first few repeats of the LRR are in close interaction with conserved motifs found in other domains, which keeps the protein in its inactive ADP state and prevents auto-activity (Slootweg et al., 2013; Wang et al., 2019a; Wang et al., 2019b). It is oftentimes the LRR that interacts (in)directly with effectors, which lifts the auto-inhibition of the NB-ARC by the LRR (Qi et al., 2012; Slootweg et al., 2013; De Oliveira et al., 2016). This recognition allows for a relaxation of the protein conformation, enabling ADP/ATP exchange, promoting oligomerization with (other) NLRs and triggering downstream immune pathways to limit pathogen spread.

Recently, cryogenic electronic microscopy (cryo-EM) studies showed that the Arabidopsis CNL HOPZ-ACTIVATED RESISTANCE 1 (ZAR1, confers resistance against *Pseudomonas syringae* (Laflamme et al., 2020), *Xanthomonas campestris* and *X. perforans* (Wang et al., 2015a; Schultink et al., 2019)) transforms upon activation from its inactive form, through an intermediate state, into a pentamer, with its N-terminal CC domain at the heart, while the C-terminal LRR domain extends outward (Wang et al., 2019a). This resistosome is structurally quite similar to the mammalian apoptosomes and inflammasomes that are formed in response to stress and trigger cell death upon activation. It is currently not known what exact downstream pathways are triggered by the formation of the ZAR1 pentamer. However, similarities of the resistosome with pore-forming proteins, as well as recent *in vivo* studies, suggests it triggers changes in Ca^+^ ion flux, eliciting other signaling responses (Bi et al., 2021). Following the discovery of the ZAR1 resistosome, two other cyro-EM studies revealed resistosome formation by the Arabidopsis TNL *RPP1* (resistance against *Peronospora parasitica*) and the *Nicotiana benthamiana* TNL *Roq1* (resistance against *Xanthomonas* sp. and *Pseudomonas* sp.) (Ma et al., 2020; Martin et al., 2020b).

The LRR domain of all three aforementioned NLRs have a typical length (ZAR1: 313 amino acids (aa), RPP1: 462, *Roq1*: 516) and number of LRR repeats (ZAR1: 13, RPP1: 21, ROQ1: 21) that form the archetype horseshoe shape (Wang et al., 2019b; Wang et al., 2019a; Ma et al., 2020; Martin et al., 2020b). The CNL *Tsw* from *Capsicum chinense* (resistance against *Tomato spotted wilt orthotospovirus*; TSWV), has an LRR domain of an estimated 1,652 aa (Kim et al., 2017), and thus belongs to the small group of NLRs with unusually large LRR domains. Typical protein domain dimensions rarely exceed 500 aa due to stability constraints (Xu and Nussinov, 1998), while these unusual LRRs range from 1,000 to 3,400 aa and contain over 30 LRR repeats. The added value of an extreme large LRR is unknown. It has been hypothesized that the LRR size (1,951 aa) of *Rps11* from soybean is linked to its ability to resistance against a broad range of *Phytophthora* sp. (Wang et al., 2021). However, (so far) *Tsw* only recognizes the effector NSs from TSWV, and its resistance can be broken with the change of 1 aa (Almási et al., 2017). The protein domains between which the (in)direct interaction of *Tsw* and NSs takes place are unknown, which leaves the function of largest part of the LRR structure unexplained. This paper discusses the structural modeling limits and various aspects of the structure-function relation conundrums of large LRRs while focusing on *Tsw*, and poses questions regarding its recognition of its effector NSs and the possible inhibition on other domains as seen in other NLRs.

## Materials and methods

### Sequence analysis of Tsw and Tsw homologs

The amino acid and coding sequences of *Tsw* (A0A1C9TCM9_CAPCH) published by Kim et al. were used to search the *C. chinense* ‘PI159236’ scaffolds and assembled genome (Chinense.v.1.2 from the Pepper Genome Platform) for the full-length sequence of *Tsw*. The amino acid sequence of Tsw was also used to search the at the time most recent version of the genome of the susceptible *C. annuum* cv. CM334 version 1.55 for the full-length non-functional variant of *tsw* (Kim et al., 2017).

Tsw homologs sharing more than 30% identity and 50% coverage were retrieved from the NLRscape database (NLRscape, 2022 https://nlrscape.biochim.ro) and analyzed with NLRscape webserver tools. These were further curated by eliminating CC/NBS incomplete and motif missing sequences, and by retrieving their secondary structure propensities and LRR repeat profiles precomputed with LRRpredictor (Martin et al., 2020a) and NLRexpress (Martin et al., 2022) from NLRscape DB. Variability profiles were raised over this cleaned Tsw family set by computing Kullback–Leibler divergence of the alignment with respect to Blosum62 background distribution using Logomaker API (Tareen and Kinney, 2020) and secondary structure consensuses were generated for graphics display.

### Large LRRs identification

A set of ~39,469 nonredundant LRR domains (90% identity cutoff) originating from plant NLR sequences were retrieved from NLRscape (NLRscape, 2022, https://nlrscape.biochim.ro) and further processed for analysis. All sequences containing annotated LRR domains at 90% redundancy were collected using NLRscape provided online selection tools. Sequence data, domain annotations, and individual LRR repeat predictions computed with LRRpredictor (Martin et al., 2020a) were retrieved from NLRscape public datasets (NLRscape, 2022). Statistical analyses of the data were performed using in-house scripts. Figures were generated using Plotly (Plotly Technologies Inc, 2015), Matplotlib (Hunter, 2007) and ETE3 (Huerta-Cepas et al., 2016) libraries.

### Homology modelling

Due to some problems that will be clarified below, homology models were built herein by two distinct methods: (1) by using the recently developed AI-based AlphaFold (Jumper et al., 2021) and (2) by using a system adapted modeling workflow that has previously been used successfully (Slootweg et al., 2013; Kozuki et al., 2017; Zhang et al., 2019). As RAM requirements grow exponentially with the sequence length, AlphaFold failed to generate a model for the long functional *C. chinense Tsw* form comprising of 2,116 aa. In addition, all models of the shorter susceptible (tsw) variant of Tsw in *C. annuum* (1,437 aa) proposed by AlphaFold were in the open ATP active state and none in the inactive ADP resting state, which seems unrealistic. Furthermore, we found that some of the delineation of LRR repeats as well as the local 3D structure in the LRR region proposed by AlphaFold is problematic. This prompted us to develop alternate models allowing to explore a larger configurational space consistent with the local sequence propensity as well as the overall architecture of the LRR domain.

The alternate models of the *Tsw*-CC, -NBS and -LRR domains were built using Modeller v10.1 (Webb and Sali, 2016) primarily based on ZAR1 ADP-bound cryo-EM conformation - PDB: 6j5w (Wang et al., 2019b). The CC and NBS domains were modelled based exclusively on the ZAR1 template as it shows the highest similarity to the *Tsw* sequence with: CC ~15% and NBS ~30% sequence identity. By contrast the LRR domain was modelled based on the repeat delineation provided by LRRpredictor (Martin et al., 2020a) using the Joint Fragment Remote Homology Modeling (JFRHM) approach (Slootweg et al., 2013) starting from: ZAR1-LRR, FLS2-LRR (PDB: 4mn8) (Sun et al., 2013) and SCHENGEN3-LRR (PDB: 6s6q) (Okuda et al., 2020). LRRpredictor indicates that the long functional form of TSW-LRR domain has 57 LRR repeats organized as follows: a starting core region of 15 repeats followed by 7 consecutive quasi-identical blocks consisting each of 6 LRR repeats. The first 10 repeats of the core region were modelled using ZAR1 as template by preserving its curvature and all its contacts with CC-NBS. The rest of the LRR domain was modeled using the other two templates depending on the local best match. To smooth out the transition between these two regions with distinct curvatures, a ‘buffer’ region consisting of repeats 10-15 was relaxed by gradually modifying the local radius using a template mixture protocol allowing the passage from the tighter ZAR1 region, r1-r10, to the broader r15-r57 rest. The C-terminal acidic tail of LRR was not covered in the model due to the lack of 3D templates and the low confidence of the secondary structure consensus within this area. The model of the shorter susceptible variant tsw was obtained using the optimized long Tsw model, as the two sequences are highly homologous (>83.6% identity). These raw models were subjected to an optimization workflow consisting of interactive rounds of energy minimization, simulating annealing and short-term Molecular Dynamics (MD) trials followed by modelling refinements. The compliance of geometric features to known 3D structures was probed using Molprobity (Williams et al., 2018). To further optimize its stability, the model was further subjected to an equilibration stage performed in explicit TIP3P water and a 0.5 nM NaCl neutral charged environment. The system was initially minimized and gradually heated from 0 to 300K and further subjected to 5 equilibration stages of gradually relaxing restraint profiles: from stronger backbone constraints in the first stage, to no constraints in the last stage. After the equilibration, the system was subjected to a long-run MD stability trial. Simulations were performed using NAMD 3.0a8 (Phillips et al., 2005; Phillips et al., 2020) and CHARMM 16m (Huang et al., 2017) forcefield using periodic boundary conditions (PBC) and Particle Mesh Edwards (PME) electrostatics with a 12Å cutoff, 10Å switch and 14Å pairlist distances for nonbonded interactions. Constant temperature (300K) and pressure (1 atm) control was attained by using the Langevin integrator and Langevin piston. Trajectory analyses were performed using VMD (Humphrey et al., 1996) and the 3D molecular graphics displayed in the figures was generated using PyMOL (The PyMOL Molecular Graphics System, Version 2.2.3 Schrödinger, LLC).

## Results

### Large LRR domains in plant NLRs

**Figure 1:**
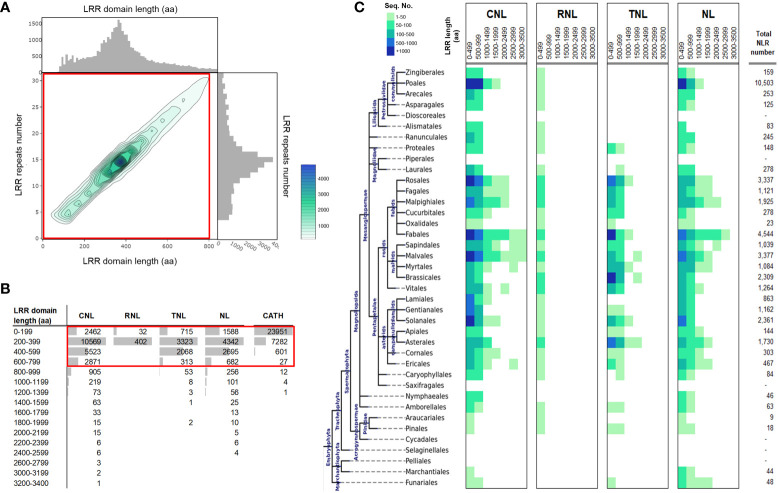
LRR domain length statistics over plant NLRs. **(A)** 2D histogram of the LRR domain length (aa) versus the corresponding number of LRR repeats. Individual 1D histograms of the LRR domain length and LRR repeats number are shown alongside the x and y axis of the 2D histogram plot. Heatmap color scale and contours legend is shown on the right side of the panel. **(B)** LRR domain length (aa) distribution in the main NLR classes: the CC (CNLs), the Resistance to Powdery Mildew 8-like domain (RPW8)) NLRs (RNLs) the Toll-interleukin receptor (TIR) (TNLs), and NLRs without an N-terminal domain (NLs). The last column (CATH) shows the domain lengths computed on experimental structural data from PDB using the annotated domains from the CATH database at 40% identity redundancy. **(C)** LRR domain length in the four main NLR classes (CNL, RNL, TNL and NL) spread across taxonomic orders. The species tree on the left shows the evolutionary relationships between plant orders and their parent clades.

To allow for the comparison of LRR domain lengths in plants, a set of ~39,469 LRRs from plant NLR sequences edged at 90% identity redundancy (NLRscape, 2022) was analyzed. Over three quarters of the retrieved LRRs span between 200 – 600 aa and are mainly comprised of less than 20 LRR repeats. The LRR length distribution peak is found at around 400 aa, corresponding to ~14-16 LRR repeats on average (**Figure 1A**). Both domain length and repeat number follow a unimodal distribution centered at 15 repeats and ~380 aa, with solid tails on both sides. The distribution is strongly right-sided skewed, with an elongated tail corresponding to large LRR domains. This roughly adds up to around 10% of LRR domains that are unusually long, with lengths of 1,000 to 3,400 aa that are organized in 30 or more repeats (**Figures 1A, B**). Large LRR domains are found mainly in CNL and NL receptors which display multiple examples of extremely large LRRs, of over 2,000 aa. By contrast, the RNL and TNL groups have far more regular LRRs (< 500 aa), with only few examples of more than 1,000 aa – all found in the TNL class. RNLs are quite rare, and hence far less represented in plants.

From a taxonomic point of view the vast majority of large LRRs originates from *Pentapetalae*, in particular from within the rosid clade: the fabids, with the bulk stemming from the *Fabales* order, and the malvids; *Sapindales, Malvales* and *Myrtales* orders. Interestingly, the *Brasicales* order in the malvids clade does not share this trend. Care should be taken in the interpretation of these results on the unequal statistical support given the uneven taxonomic distribution of NLRs in the database (shown in **Figure 1C**, last column).

Such large dimensions of LRR domains are puzzling as they are far larger than typical protein domains, which rarely exceed 500 aa due to stability problems (Xu and Nussinov, 1998). For instance, at 40% identity redundancy, 99.8% of the domains in the CATH database (Sillitoe et al., 2021) are shorter than 600 aa, with only 5 examples larger than 1,000 aa out of a total of 31,878, which is less than 0.02%. (**Figure 1B**, CATH column). These large dimensions are favored by the loosely and modular solenoidal LRR architecture, which, in contrast to the more globular architectures, offer flexibility and elasticity to the overall fold (Kobe and Kajava, 2001). In addition, large continuous LRR horseshoes are in some cases weakened or disrupted by relatively long insertions incompatible with the LRR fold, which potentially break the LRR domain into two or more sub-regions.

The manner in which such LRR subregions would fold with respect to each other is another still open-ended question. Shorter linkers between LRR fragments generally tend to maintain the overall horseshoe shape of the domain with minor disruptions. By contrast, longer linkers that allow for rotations and translation of the downstream LRR elements might generate a wide range of overall potential conformations – from a perfect fusion of consecutive LRR fragments, with large insertion ‘isles’ as seen in some X-ray crystal structures such as the extracellular BRI1 (Hohmann et al., 2018) or PSKR1 (Wang et al., 2015b), to even a potential side-to-side LRR-LRR fragment clustering – similar to those seen in some protein-protein interactions. In assessing the LRR domain continuity, a special indicator of the local LRR stability is the embedment of the L-x-x-L-x-L pattern into a larger pattern L-x-x-(L-x-x-L-x-L)-x-x-N/C (Kajava, 1998) that provides further stability and structural reinforcement.

Large LRR domains are also difficult to explain from a functional point of view. Experimental data, from both solved 3D structures and *in vivo* experiments, indicate that in a large and diverse set of NLR examples interactions of the LRR with the rest of NLR domains (CC, TIR, NBS) primarily take place within the first four to eight LRR repeats (Qi et al., 2012; Slootweg et al., 2013). In ZAR1 cryo-EM structures, the LRR-CC interaction occurs within the first 4 LRR repeats, while the LRR-NB-ARC interaction is consolidated mainly within the first 8 LRR repeats – in tight contact with the ARC2 subdomain of NB-ARC (Wang et al., 2019a; Wang et al., 2019b). Sparse contacts were also shown to shape up between the further five LRR repeats and the beta-turn-beta loop structure of the ARC2 domain. All these interdomain contacts are critical for a proper functioning of these receptors, as shown by swap experiments between sections of the LRR domain of different *R* genes. Compelling experimental data demonstrate the importance of the compatibility between the first LRR repeats and the rest of the NLR (Qi et al., 2012; Slootweg et al., 2013; Slootweg et al., 2017; Slootweg et al., 2018). For instance, LRR swaps between potato homologs *Rx1* and *Gpa2*, which despite their 88% sequence identity recognize unrelated pathogens, showed that successful phenotypic LRR exchanges were only obtained when preserving the original CC-NBS-first 3 LRR repeats, while the fusion of the foreign rest of the LRR domain converts the pathogen recognition (Slootweg et al., 2013; Slootweg et al., 2017; Slootweg et al., 2018).

The strong preference for 10-16 LRR repeats observed within the dataset of LRR domains (**Figure 1A**) is consistent with the existing experimental and structural data, yet it leaves a very large portion of the LRR structure unexplained in cases of extremely large LRR domains.

### Tsw homologs group

Starting from the Tsw sequence from *C. chinense*, a set of distant homologs at above 30% identity with Tsw were retrieved using the NLRscape tools (NLRscape, 2022). The set was trimmed by eliminating the highly redundant sequences by imposing a threshold of 90% identity between the identified homologs obtaining a set of 200 sequences, which we further refer to as the extended homologs group. Based on the identity matrix of the alignment, a dense group emerges, corresponding to a set of close homologs of the *Capsicum* Tsw which consists of ~32 nonredundant sequences, which share between 55-90% identity with Tsw on the full-length protein and above 70% on the NBS domain span. Outside this group, the remaining homologs in the extended set share less than 40% identity with Tsw on the overall sequence and below 50% on the NBS domain span, generating a neat separation.

The close homologs are all part of the Solanaceae family, specifically of the *Capsicum*, *Solanum* and *Nicotiana* genera, while the more distant homologs show a broader taxonomic spread primarily across various taxa orders of the malvids and rosids clades (**Supplementary File 1A**). As expected, the close homologs of Tsw displays multiple highly conserved regions, some of which are also highly conserved in the extended homologs set (**Supplementary File 1B, C**).

### Tsw 3D models

**Figure 2:**
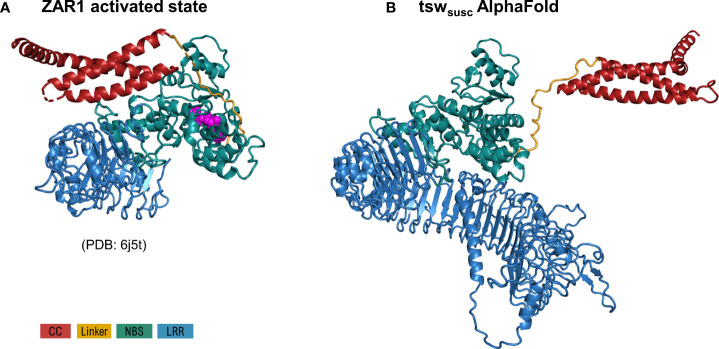
Comparison of the overall model of the susceptible tsw from *C annuum* **(A)**, as proposed by AlphaFold in most likely its activated ATP state, with the ZAR1 cryo-EM structure **(B)** in the same state (PDB: 6j5t). Configurations correspond to the superposition of NBS and the first 8 LRR repeats in both structures. As seen, the solution proposed by AF for the CC domain orientation is strikingly divergent to that experimentally determined by Cryo-EM in Zar1. Domain color code is indicated in the figure legend.

**Figure 3:**
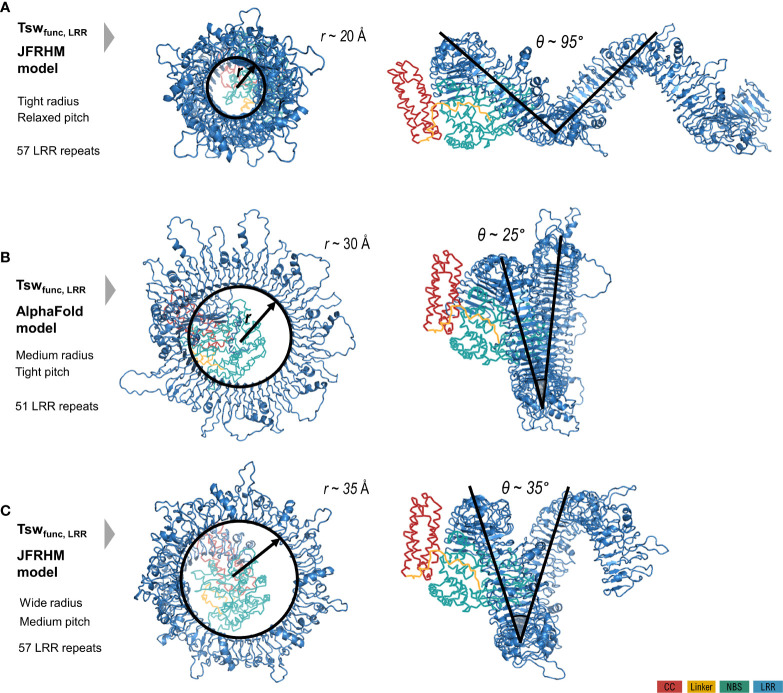
Limiting scenarios of the LRR domain structure of the functional Tsw from *C chinense* (1580 aa) with respect to the CC/NBS configuration in resting ADP state modeled starting from Zar1. LRR model as **(B)** proposed by AlphaFold automation; **(A, C)** proposed by the adapted JFRHM LRR model in two limiting scenarios of tight/relaxed radius and pitch. Domain color code is indicated in the figure legend.

As specified under methods, AlphaFold automatic modelling fails for the long 2,116 aa functional version of Tsw. Additionally, the AlphaFold model does not account for the ADP resting state of the shorter susceptible 1,438 aa variant (**Figure 2**), nor does it unambiguously match with the motif delineation proposed by LRRpredictor. This prompted us to build additional system adapted models to overcome these limitations. Such adapted models furthermore allowed us to explore an opposed limiting scenario, which in conjunction with the AlphaFold model, defines the range in which we could expect such extensive LRR domain structures to stand. While the LRR curvature proposed by AlphaFold is wide – ~60 Å in diameter with a ~25° pitch of the supercoiled solenoid – the adapted models explore a wider range of diameter (40-70 Å), pitch angles (35-95°) of the supercoil bending and optimized repeat delineation. In a conformation with a wide radius, tight pitch and only 51 repeats, such as the one proposed by the AlphaFold model, the curvature translates into ~1.5 supercoiled turns. This is compared in **Figure 3** with alternate Tsw-LRR models of 57 repeats in two extreme scenarios of relaxed vs. tight radius and pitch. These suggest that pitches narrower than ~25-35° potentially imply intra-domain interactions between consecutive turns of the LRR domain. It is therefore more probable to find the real structure standing and flexing in between these two extreme limits.

**Figure 4:**
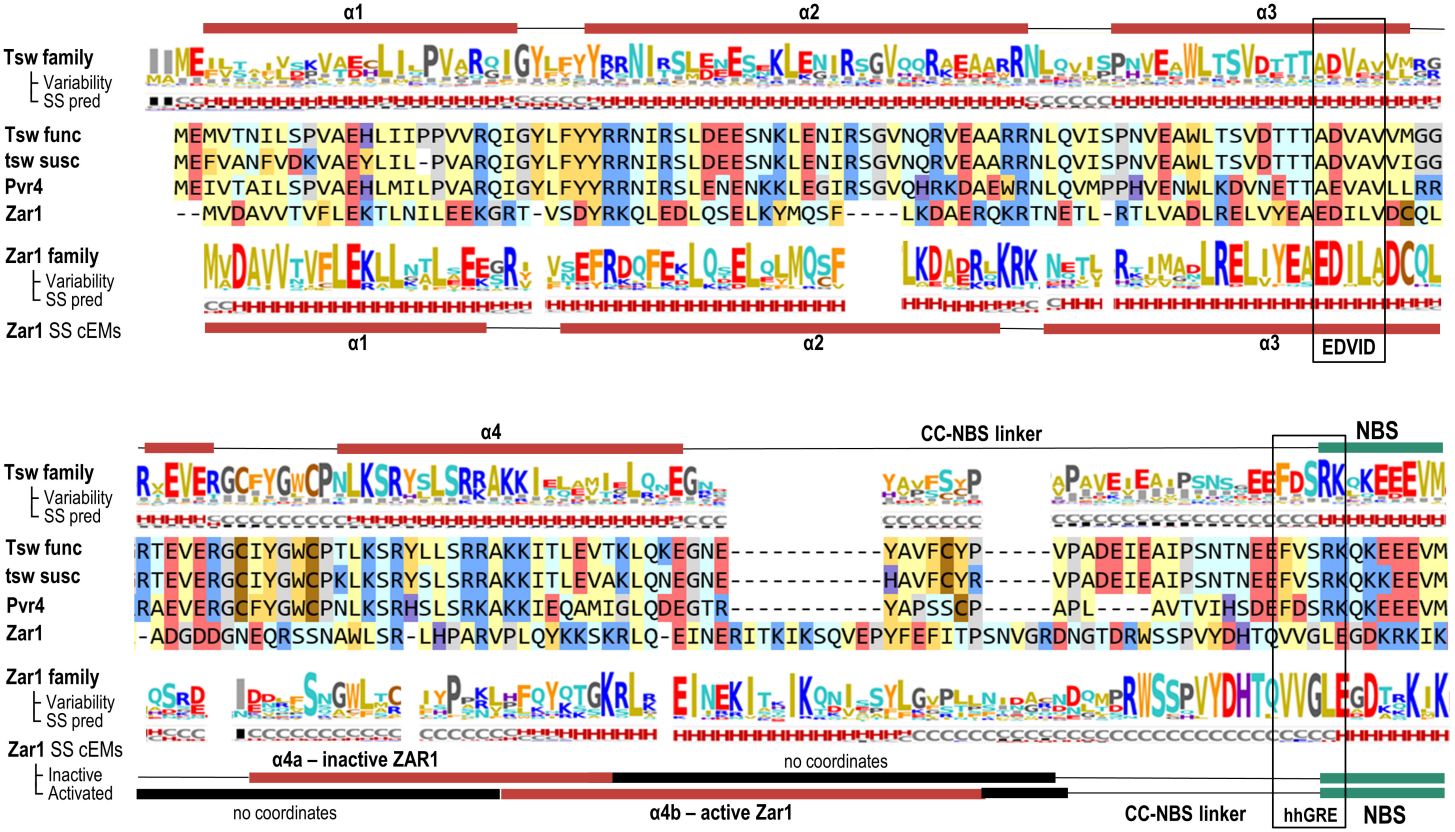
Coiled Coil (CC) domain: Sequence to structure mapping of *C. chinense* and *C. annuum* Tsw to the *A. thaliana* ZAR1 template. The helical predicted structure is shown above the alignment. Variability and secondary structure (SS) prediction consensus lines are computed on the Tsw and ZAR1 homologous families at a 50% identity threshold using NLRscape (NLRscape, 2022 at https://nlrscape.biochim.ro). Variability is represented as relative entropy with respect to the background distribution - taller letters correspond to more conserved positions in the family. Secondary structure line reads: H – helix (red), E – extended (blue), C – coil (grey) with letter heights proportional to the percentage in the alignment. Helical structure assignments in cryo-EM inactive form (PDB: 6j5w) and oligomeric ZAR1 form (PDB: 6j5t) are shown below the alignment. Amino acids are colored according to their physical-chemical properties as follows: yellow - aliphatic hydrophobic; orange – aromatic; brown – cysteine; red - acidic negatively charged; blue - basic positively charged; light blue - polar neutral charge; purple - histidine; gray - glycine and proline.

The Tsw-CC domain, aa 1-129, only displays remote homology to solved CC structures. Of these, ZAR1-CC is the closest with 13-17% identity depending on the alignment method. Secondary structure predictions indicate the presence of four helical segments in +/-1 turn agreement to ZAR1. Moreover, all four helices have an amphipathic pattern consistent with a 4-helical bundle fold with the hydrophobic regions hidden inside the bundle. As in ZAR1, the Tsw-CC N-terminal region of the first helical stretch, α1, is overall hydrophobic, which might protrude the membrane in the active state similarly to that in ZAR1 (**Figure 4**). The next two helical segments α2 and α3 have an even higher similarity to ZAR1. Tsw also contains a partial EDVID motif (aa 81- 85: “ADVAV”) known to be involved in CC-LRR interaction. However, this is not a shared feature, as the third helix (α3) shows a significant variability even in the 32 close homologs of 50-90% identity (**Figure 4**). The absence of a conservation pressure within this region might indicate that the CC-LRR interaction pattern of Tsw family might be distinct from ZAR1.

Interestingly, the Tsw family displays highly conserved aromatic residues in positions equivalent to both the α1-α2 and the α3-α4 interconnecting loops in ZAR1. These two loops are close in distance in the 4-helical bundle structure, forming a contiguous surface exposed to the solvent. The location and degree of conservation of this patch might indicate a potential protein-protein interaction hotspot.

Lastly, α4 has the lowest homology with known templates, including ZAR1. In ZAR1 the predicted α4 is very long and was shown by cryo-EM to be unstable, as it folded differently with respect to α1, α2, and α3 in the resting and active state. While in the resting ADP state, only the first part of α4, named here α4a, closes the 4-helical bundle letting the second α4b part disordered. In the active ATP state this disordered region of α4b freezes, forming the four helical bundle and letting α4a disordered. By contrast, in Tsw the predicted α4 is significantly shorter and continues with a highly variable segment with linker-like propensities such as in the Rx1 template.

**Figure 5:**
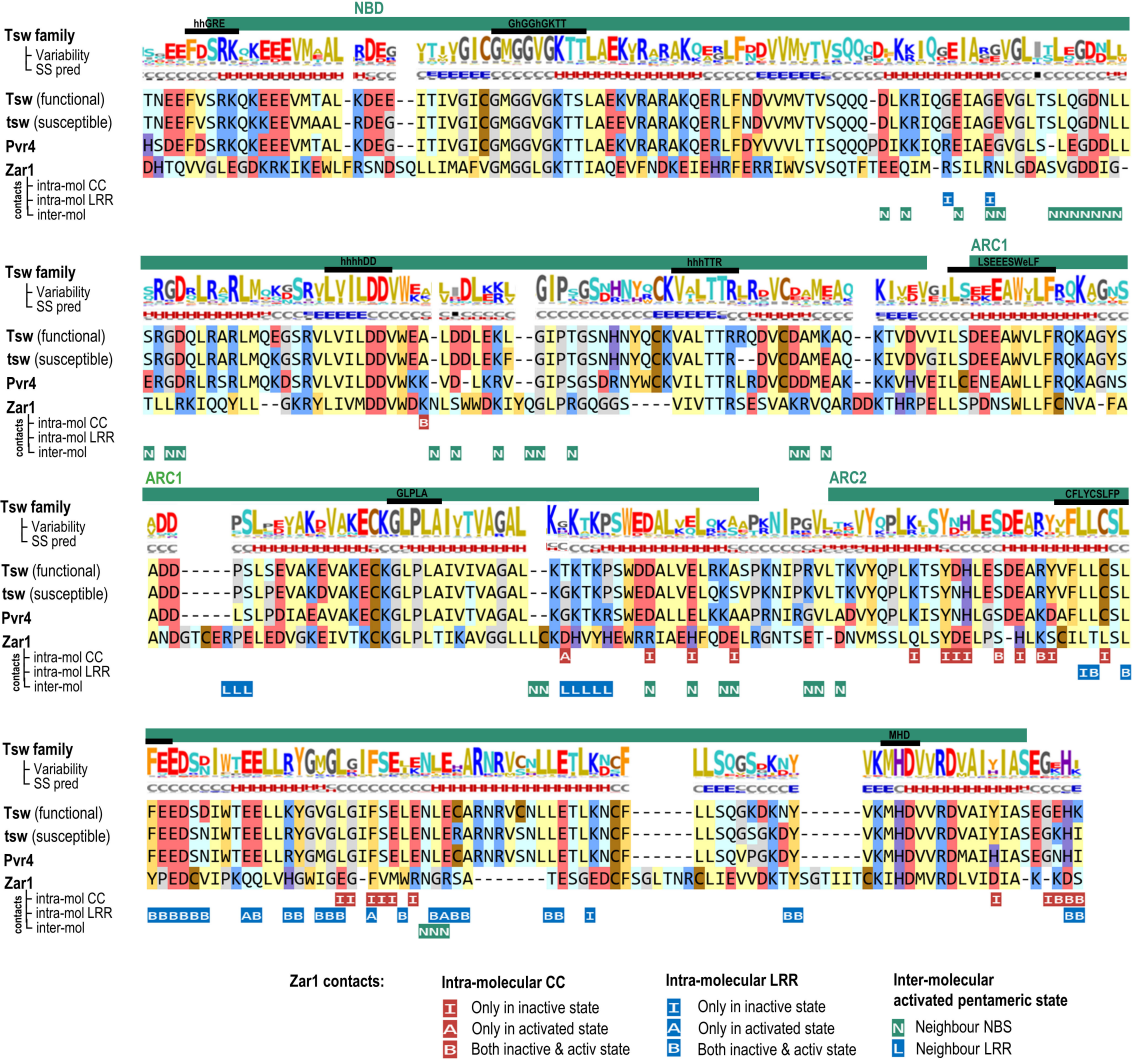
NBS: Sequence to structure mapping of *C. chinense* and *C. annuum* Tsw to *A. thaliana* ZAR1 template. Sequence motifs and subdomains are annotated above the alignments. The variability and secondary structure (SS) prediction consensuses are computed and displayed as described in the **Figure 4** caption. Below the alignment are mapped the intra and inter-molecular contacts (under 5 Å) of ZAR1 cryo-EM structure in resting and activated conformations (6j5w, 6j5t).

The Tsw-NBS domain (aa 158-495) shares 30% identity to ZAR1 and it preserves all the functionally significant regions: the P-loop, Walker-B, RNBS motifs A through D, the GLPL motif, and the MHD motifs (**Figure 5**). By contrast, the N-terminal entry into the NBS diverges from the common VG motif (Wróblewski et al., 2018), which in Tsw, both in close and extended homologs groups, is of type “eeFdSR” (where lower case show the consensus amino acids with lower conservation). Not all, but only some of the intra- and inter-molecular contacts seen in ZAR1 cryo-EMs, are conserved in Tsw. As seen for other R proteins, for instance in Sw-5a and -b (De Oliveira et al., 2016), point mutations between the functional and susceptible variants generate changes in salt bridge patterns at the NBS-LRR interface such as Tsw: D420-K540 changed to N417-K537 in tsw; or Tsw: C444-E660 changed to R441-E657 in tsw.

**Figure 6:**
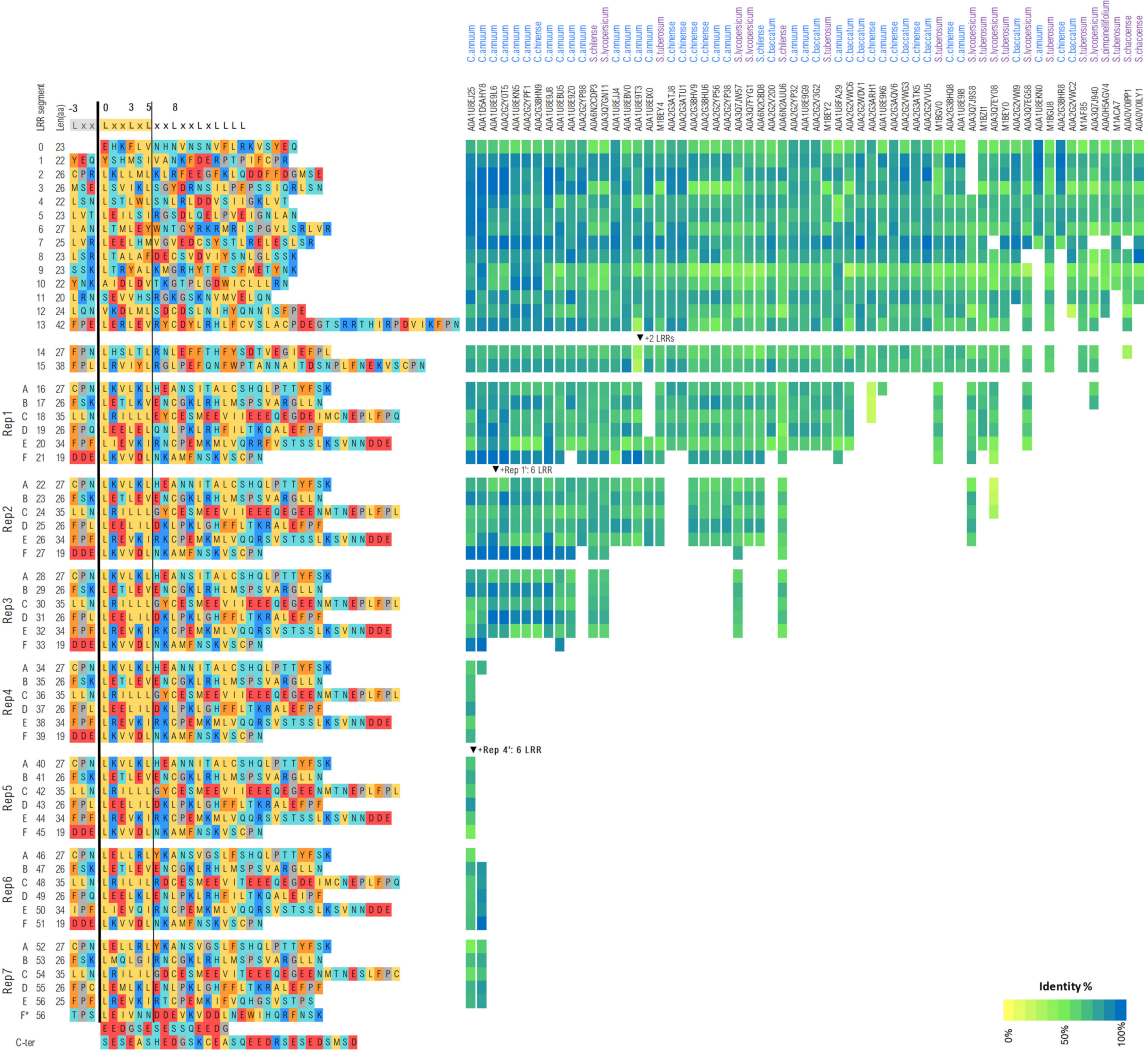
LRR repeat delineation of Tsw from *C. chinense*. The right hand side of the figure shows Tsw’s closest homologs from *Capsicum* (blue) and *Solanaceae* (purple) families alongside with the conservation variations across each LRR repeat.

The LRR domain of the functional *C. chinense* Tsw variant spans over ~1,550 residues (aa 511-2,067). LRRpredictor delineates 57 LRR repeats, with several lower probability motifs discussed in detail below. Briefly, starting from the N-terminal side, the LRR domain is structured in a core section of 15 LRR repeats (aa 511-901), followed by seven consecutive quasi-identical blocks each consisting of six LRR repeats named A-F. The last repeat, 7-F, is incomplete and followed by a highly acidic tail with high propensity for an intrinsically disordered structure (aa 2,068-2,116) (**Figure 6**).

In Tsw, the NB-ARC-LRR linker corresponding to the region between the end of the ARC2 subdomain (aa 495) and the first proper LRR motif (aa 512 - YSHMSI) is nearly double in size to its equivalent region in ZAR1: 17 aa vs. 9 aa. In the cryo-EM structure of ZAR1, this 9 aa linker folds as an improper LRR repeat that is loosely attached to the rest of the LRR domain. In Tsw, its 17 aa equivalent has notable similarities with proper LRR repeats, both in length and secondary structure propensity. It was therefore modeled as such and referred herein as the LRR 0 repeat (**Figure 6**).

In Tsw, the first seven LRR repeats, with which interactions with CC and NBS are expected to occur, show significant similarities with ZAR1. LRR motif predictions become problematic in the region aa 740-780. The motif probability (aa 761 - SEVVHS) is not very high here, thus this could be modeled either as a single long 44 aa repeat, or as two shorter repeats. Such a motif has a very low LRRpredictor probability. However, the propensity of this stretch for extended secondary structure and forming beta sheets is high. Hence, this was locked as a proper motif in both AlphaFold and adapted-JFRHM models.

The AlphaFold oddly models the orientation of LRR 9 in reverse, i.e. with its dorsal side oriented towards the inside of the horseshoe. None of the structurally solved LRR domains contains such an odd repeat orientation. Moreover, this orientation is inconsistent with overall NLR architecture in the resting ADP state, as such a reverse repeat would clash with the NBS domain. Therefore, most likely the local solution adopted by AlphaFold for LRR 9 is an artefact derived from fusing two templates and was corrected in the adapted-JFRHM model.

In each of the following seven blocks, LRRpredictor identifies six repeats termed herein A-F. This in contrast to AlphaFold, which merges repeats E and F in a single 53 aa strand. In our A-F delineation, all motifs on the ventral side of the horseshoe are of one of the following extended types: L-x-x-(L-x-L-x-x-L)-x-x-L-x-x/L-L-L-x/L, suggesting a structural reinforcement that was taken into consideration in the adapted models. Interestingly, all the A-F repeats display in addition a stretch of two to three consecutive hydrophobic residues on the dorsal side of the horseshoe in the 14–18 aa region of each repeat. This could potentially form a continuous hydrophobic ‘band’ over the entire structure.

The susceptible variant tsw from *C. annuum* lacks blocks 3–6, while the core section of block 1 and 7 follows the same LRR organization as the functional variant with only minor sequence variations (94.0% identity at nucleotide level). Apart from the missing LRR blocks, the last 23 aa at the C-terminal end – within the second half of the acidic tail – of tsw has a completely different, highly positively charged, aa sequence due to a frameshift resulting from a 1 nucleotide deletion. This makes a striking distinction between the functional vs susceptible variant, *i.e*. the C-terminal tail of Tsw being entirely acidic, while the tail of tsw is half acidic, half basic. Such differences could be responsible for the loss of its functionality as a resistance gene.

Interestingly, when inspecting close homologs of Tsw within the *Solanaceae* clade, the lengths of the LRR domains varies significantly, with most homologs not exceeding two or three repetitive blocks (**Figure 6**). Common to all, the core region of the LRR domain shows a higher degree of conservation within the N-terminal region (LRR 1-8), which is consistent with the region’s expected involvement in NBS interaction.

Furthermore, the central C repeat of each repetitive block is highly negatively charged. This creates a noticeable repetitive pattern in 3D, and in addition makes each module slightly negatively charged. Overall, this adds up to an extreme -32 total net charge on the LRR domain span. When also considering the net charges of the CC (+6), CC-NBS linker (-7), NBS (-7), and the acidic C-terminal tail (-17), the total net charge of the overall protein becomes -57, which has to be compensated by the environment, or the folding of the protein. Tsw’s total net charge is quite extreme in comparison to other R proteins, for which it generally ranges around -10 to +10. When compensated for by length, the total net charge remains below average.

While both the AlphaFold and the JFRHM adapted models display the LRR domain as a continuous solenoid supercoil, it cannot be excluded that the continuity of the LRR domain might be disrupted or altered at the less reinforced regions of the LRR pattern due to the molecular environment or on the large biological time scale.

### Inter-domain interactions

AlphaFold only models Tsw starting from the ZAR1 ATP active state. Moreover, the five best AlphaFold models display the CC domain in a completely different configuration and opposite orientation to that seen in ZAR1-ATP active state (**Figure 2**). This local modeling solution adopted by AlphaFold results in a complete disruption of the interface between CC-NBS and CC-LRR as that seen in ZAR-ATP state (**Figure 2**), suggesting that the amino acids at the predicted interface are not compatible with a CC-NBS and CC-LRR contact state.

**Figure 7:**
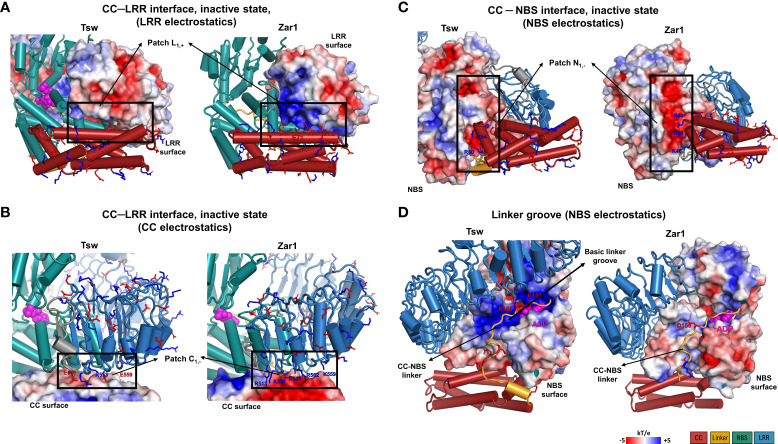
Electrostatic properties of the interdomain contact surfaces mapped on the functional Tsw adapted JFRHM model and ZAR1 inactive ADP-binding cryo-EM structure (PDB: 6j5w). **(A, B)** CC-LRR interface electrostatic potential mapped on **(A)** the LRR and **(B)** CC domains. **(C, D)** Electrostatic potential surface of NBS domain at the contact interface with **(C)** the CC domain and **(D)** the CC-NBS linker. Negatively (red) and positively (blue) charged residues located at inter-domain interfaces (dotted line boxes) are labeled and displayed in stick representation. Electrostatic potential scale and domain color code are indicated in the figure legend.

To further investigate this incompatibility, we analyzed the corresponding CC-NBS and CC-LRR interface on the JFRHM models generated starting from the inactive ADP conformation of ZAR1. As can be seen in **Figure 7**, indeed the amino acid properties at the CC-LRR interface do not match perfectly with those seen in the ZAR1 template, suggesting that the interaction between these two domains might be different in Tsw. In ZAR1, the CC-LRR interface is shared between the inactive and active conformations and has a strong electrostatic nature (**Figures 7A, B**) with the CC acidic EDVID motif area in α3 being complemented by a proportionally basic patch on the lower side of the first four LRR repeats. On the other hand, in Tsw the interface is neutral, suggesting a far weaker affinity.

By contrast, the CC-NBS interface shares a very similar electrostatic profile with that seen in ZAR1 in its resting ADP state. The NBS part of the interface consists of a negatively charged patch N_1,-_ (ARC1 end, ARC2 beginning) in contact with the positively charged region at the end of α3 and the outset of the α4 helix of the CC domain (**Figure 7C**).

Focusing now on the groove of formed across the NBS domain accommodating the CC-NBS linker in the JFRHM model of Tsw built starting from the ZAR1 inactive ADP template, the compatibility between the linker sequence and the NBS groove is even higher in Tsw than in ZAR1 due to a striking opposite charge matching (**Figure 7D**). In Tsw, the NBS groove is highly positively charged and the linker displays an increased net negative charge of -5 (aa 140-156), whereas the interface in ZAR1 has a more homogenous neutral charge. Together, these findings suggest that Tsw shares only partially interdomain interface profiles with ZAR1, implying that the interaction between domains might be different from the ones seen in ZAR1 during activation.

## Conclusion and reflections

The activation of the 10% of NLRs with ≥1,000 aa-long LRRs have scarcely been elucidated. The modelled structures of the resistant and susceptible Tsw proteins provide a glimpse into the intricate resistance pathways in plants, although the exact function of the large LRRs remains a conundrum. Cryo-EM studies have only quite recently revealed multimeric resistosome formation from activated monomeric NLR proteins (Wang et al., 2019a; Ma et al., 2020; Martin et al., 2020b). It is unknown whether the formation of resistosomes is a general feature of NLRs, or an anomaly amongst NLRs. Their resemblance to apoptosomes and inflammosomes, however, provides support for these oligomeric structures as a generic feature of NLRs. For quite a number of NLR proteins researchers have demonstrated self-association and the formation of dimers and oligomers (Bernoux et al., 2011; Maekawa et al., 2011; Casey et al., 2016; Zhao et al., 2021). From such configurations it is only a small step towards an activated pentameric resistosome. The question therefore arises whether these dimers and oligomers are involved in activities that are needed prior to further formation into a resistosome. All three currently confirmed resistosomes are formed by NLRs with an average LRR length, thus it will be interesting to see whether NLRs with long LRRs like Tsw are also capable of such formations. And if they are, whether they self-associate, or whether Tsw for instance requires assistance from helper NLRs for oligomerization and activation. A 3D protein model of Tsw has been proposed based on all currently available modelling tools, although these are not always suited for such large proteins. Without cryo-EM structures of Tsw, no definitive conclusions can be drawn. As it is likely that cryo-EM structures will be resolved for NLRs with long LRRs such as Tsw, this subject might be resolved in the future. However, preliminary conclusions can still be drawn based on our proposed 3D model.

Upon seeing the highly unusual helical turn made by the LRR in functional Tsw, questions arise regarding the interaction of the LRR with other proteins. Are NLRs with long LRRs capable of directly interacting with effectors, or indirectly with a guardee/decoy protein? Is there a common denominator between these unusual R proteins, potentially indicating a shared protein interaction? Furthermore, one wonders whether the long LRR is crucial for the interaction, or whether as with typical NLRs the C-terminal end is the determinant for the interaction. The helical turn creates a large cavity in the LRR domain, which potentially may be indictive of the size and shape of its interactor, as is the case for CNL protein CYR1 from mungbean (normal length LRR), and its effector, the coat protein of *Mungbean yellow mosaic India virus* (MYMIV). *In silico* models revealed a niche formed by CYR1 in which MYMIV-CP fitted neatly upon their interaction (Maiti et al., 2012). Modeling attempts have been made for NSs (Olaya et al., 2019). However, when comparing Tsw and NSs, it is important to bear in mind that there is little to no resemblance in sequence between proteins with 3D structures confirmed by cryo-EM and TSWV NSs. The increasingly accurate protein structure prediction programs are steadily modeling many potential interaction partners and could in the future be used to support other findings.

The 3D model of Tsw allows for structure-informed decisions regarding future functionality experiments. The similarity between α1-α2 and α3-α4 in ZAR1 and Tsw might indicate a possible shared protein interaction spot, while the low conservation of α3 in the Tsw family could denote a potentially divergent interaction between CC and LRR in this family. Mutations in this region might therefore shed light on this possibility, while truncations in the LRR domain could aid in uncovering the function of the long LRR domain, and the need for the extra helical turn, as well as determining the necessity of the C-terminal tail in the recognition of NSs. Similar experiments have been performed for Pvr4, a homolog of Tsw from *C. annuum* that confers resistance against potyviruses (Kim et al., 2017), where removal of three out of six LRR repeats triggered auto-immunity (Kim et al., 2018). Chimeras of the non-functional (susceptible) tsw and functional Tsw in which LRR domains are swapped/added/deleted would likewise allow for highly interesting functional studies. Similarly, a C-terminal end swap between Tsw and Pvr4 would determine whether this segment is responsible for pathogen recognition, much alike Gpa2 and Rx1 (Slootweg et al., 2017). Furthermore, as downstream signaling upon Pvr4 activation may function due to its CC_NO-EDVID_ domain, a CC swap or mutation of the EDVID-like domain in Tsw would allow for potentially interesting observations. Incorporation of the hypotheses mentioned here in future research would potentially enhance our understanding of the roles of long LRRs, as well as other domains of Tsw, in both pathogen recognition and downstream immune pathways.

## Data availability statement

The original contributions presented in the study are included in the article/**Supplementary Material**. Further inquiries can be directed to the corresponding authors.

## Author contributions

Research design and conceptualization: RK and A-JP. Computational analysis, methodology, investigation, data collection and analysis, visualisation: IG and EM. Writing – original draft preparation: IG and EM. Writing – review and editing: All authors. Supervision, project administration and funding acquisition: RK and A-JP. All authors contributed to the article and approved the submitted version.

## Funding

This work is supported by the Netherlands Organization of Scientific Research (NWO; grant ALWGR.2015.3) (IG and RK) and the Romanian Academy programs 1 & 2 of IBAR (EM and A-JP).

## Conflict of interest

The authors declare that the research was conducted in the absence of any commercial or financial relationships that could be construed as a potential conflict of interest.

## Publisher’s note

All claims expressed in this article are solely those of the authors and do not necessarily represent those of their affiliated organizations, or those of the publisher, the editors and the reviewers. Any product that may be evaluated in this article, or claim that may be made by its manufacturer, is not guaranteed or endorsed by the publisher.

## References

[B1] Almási A. Nemes K. Csömör Z. Tóbiás I. Palkovics L. Salánki K. (2017). A single point mutation in tomato spotted wilt virus NSs protein is sufficient to overcome tsw-gene-mediated resistance in pepper. J. Gen. Virol. 98, 1521–1525. doi: 10.1099/jgv.0.000798 28631603

[B2] The PyMOL molecular graphics system, version 2.2.3 (Schrödinger, LLC).

[B3] Bernoux M. Ve T. Williams S. Warren C. Hatters D. Valkov E.. (2011). Structural and functional analysis of a plant resistance protein TIR domain reveals interfaces for self-association, signaling, and autoregulation. Cell Host Microbe 9, 200–211. doi: 10.1016/j.chom.2011.02.009 21402359 PMC3142617

[B4] Bi G. Su M. Li N. Liang Y. Dang S. Xu J.. (2021). The ZAR1 resistosome is a calcium-permeable channel triggering plant immune signaling. Cell 184, 3528–3541.e12. doi: 10.1016/j.cell.2021.05.003 33984278

[B5] Casey L. W. Lavrencic P. Bentham A. R. Cesari S. Ericsson D. J. Croll T.. (2016). The CC domain structure from the wheat stem rust resistance protein Sr33 challenges paradigms for dimerization in plant NLR proteins. Proc. Natl. Acad. Sci. 113, 12856–12861. doi: 10.1073/pnas.1609922113 27791121 PMC5111715

[B6] De Oliveira A. S. Koolhaas I. Boiteux L. S. Caldararu O. F. Petrescu A.-J. Oliveira Resende R.. (2016). Cell death triggering and effector recognition by sw-5 SD-CNL proteins from resistant and susceptible tomato isolines to tomato spotted wilt virus. Mol. Plant Pathol. 17, 1442–1454. doi: 10.1111/mpp.12439 27271212 PMC6638320

[B7] Duxbury Z. Wu C.-H. Ding P. (2021). A comparative overview of the intracellular guardians of plants and animals: NLRs in innate immunity and beyond. Annu. Rev. Plant Biol. 72, 155–184. doi: 10.1146/annurev-arplant-080620-104948 33689400

[B8] Hohmann U. Santiago J. Nicolet J. Olsson V. Spiga F. M. Hothorn L. A.. (2018). Mechanistic basis for the activation of plant membrane receptor kinases by SERK-family coreceptors. Proc. Natl. Acad. Sci. U.S.A. 115, 3488–3493. doi: 10.1073/pnas.1714972115 29531026 PMC5879659

[B9] Huang J. Rauscher S. Nawrocki G. Ran T. Feig M. de Groot B. L.. (2017). CHARMM36m: an improved force field for folded and intrinsically disordered proteins. Nat. Methods 14, 71–73. doi: 10.1038/nmeth.4067 27819658 PMC5199616

[B10] Huerta-Cepas J. Serra F. Bork P. (2016). ETE 3: Reconstruction, analysis, and visualization of phylogenomic data. Mol. Biol. Evol. 33, 1635–1638. doi: 10.1093/molbev/msw046 26921390 PMC4868116

[B11] Humphrey W. Dalke A. Schulten K. (1996). VMD: Visual molecular dynamics. J. Mol. Graph 14, 33–38. doi: 10.1016/0263-7855(96)00018-5 8744570

[B12] Hunter J. D. (2007). Matplotlib: A 2D graphics environment. Comput. Sci. Eng. 9, 90–95. doi: 10.1109/MCSE.2007.55

[B13] Jones J. D. G. G. Dangl J. L. (2006). The plant immune system. Nature 444, 323–329. doi: 10.1038/nature05286 17108957

[B14] Jumper J. Evans R. Pritzel A. Green T. Figurnov M. Ronneberger O.. (2021). Highly accurate protein structure prediction with AlphaFold. Nat 596, 583–589. doi: 10.1038/s41586-021-03819-2 PMC8371605 34265844

[B15] Kajava A. V. (1998). Structural diversity of leucine-rich repeat proteins 1 1Edited by f. Cohen. J. Mol. Biol. 277, 519–527. doi: 10.1006/jmbi.1998.1643 9533877

[B16] Kim S. Kang W. Huy H. N. Yeom S. An J. Kim S.. (2017). Divergent evolution of multiple virus-resistance genes from a progenitor in capsicum spp. New Phytol. 213, 886–899. doi: 10.1111/nph.14177 27612097

[B17] Kim S.-B. B. Lee H.-Y. Y. Choi E.-H. H. Park E. Kim J.-H. H. Moon K.-B. B.. (2018). The coiled-coil and leucine-rich repeat domain of the potyvirus resistance protein Pvr4 has a distinct role in signaling and pathogen recognition. Mol. Plant-Microbe Interact. 31, 906–913. doi: 10.1094/MPMI-12-17-0313-R 29663867

[B18] Kobe B. Kajava A. V. (2001). The leucine-rich repeat as a protein recognition motif. Curr. Opin. Struct. Biol. 11, 725–732. doi: 10.1016/S0959-440X(01)00266-4 11751054

[B19] Kozuki T. Chikamori K. Surleac M. D. Micluta M. A. Petrescu A. J. Norris E. J.. (2017). Roles of the c-terminal domains of topoisomerase IIα and topoisomerase IIβ in regulation of the decatenation checkpoint. Nucleic Acids Res. 45, 5995–6010. doi: 10.1093/nar/gkx325 28472494 PMC5449615

[B20] Laflamme B. Dillon M. M. Martel A. Almeida R. N. D. Desveaux D. Guttman D. S. (2020). The pan-genome effector-triggered immunity landscape of a host-pathogen interaction. Science 367, 763–768. doi: 10.1126/science.aax4079 32054757

[B21] Maekawa T. Cheng W. Spiridon L. N. Töller A. Lukasik E. Saijo Y.. (2011). Coiled-coil domain-dependent homodimerization of intracellular barley immune receptors defines a minimal functional module for triggering cell death. Cell Host Microbe 9, 187–199. doi: 10.1016/j.chom.2011.02.008 21402358

[B22] Maiti S. Paul S. Pal A. (2012). Isolation, characterization, and structure analysis of a non-TIR-NBS-LRR encoding candidate gene from MYMIV-resistant vigna mungo. Mol. Biotechnol. 52, 217–233. doi: 10.1007/s12033-011-9488-1 22205242

[B23] Ma S. Lapin D. Liu L. Sun Y. Song W. Zhang X.. (2020). Direct pathogen-induced assembly of an NLR immune receptor complex to form a holoenzyme. Science 370 (6521), eabe3069. doi: 10.1126/science.abe3069 33273071

[B24] Martin R. Qi T. Zhang H. Liu F. King M. Toth C.. (2020b). Structure of the activated ROQ1 resistosome directly recognizing the pathogen effector XopQ. Science 370, 1–21. doi: 10.1126/science.abd9993 PMC7995448 33273074

[B25] Martin E. C. Spiridon L. Goverse A. Petrescu A.-J. (2022). NLRexpress - a bundle of machine learning motif predictors - reveals motif stability underlying plant NLRs diversity. Front. Plant Sci. doi: 10.3389/fpls.2022.975888 PMC9519389 36186050

[B26] Martin E. C. Sukarta O. C. A. A. Spiridon L. Grigore L. G. Constantinescu V. Tacutu R.. (2020a). LRRpredictor–a new LRR motif detection method for irregular motifs of plant NLR proteins using an ensemble of classifiers. Genes (Basel) 11, 286. doi: 10.3390/genes11030286 PMC7140858 32182725

[B27] NLRscape (2022) NLRscape - a plant NLR atlas. Available at: https://nlrscape.biochim.ro (Accessed March 15, 2022).

[B28] Okuda S. Fujita S. Moretti A. Hohmann U. Doblas V. G. Ma Y.. (2020). Molecular mechanism for the recognition of sequence-divergent CIF peptides by the plant receptor kinases GSO1/SGN3 and GSO2. Proc. Natl. Acad. Sci. 117, 2693–2703. doi: 10.1073/pnas.1911553117 31964818 PMC7007523

[B29] Olaya C. Adhikari B. Raikhy G. Cheng J. Pappu H. R. (2019). Identification and localization of tospovirus genus-wide conserved residues in 3D models of the nucleocapsid and the silencing suppressor proteins. Virol. J. 16, 7. doi: 10.1186/s12985-018-1106-4 30634979 PMC6330412

[B30] Phillips J. C. Braun R. Wang W. Gumbart J. Tajkhorshid E. Villa E.. (2005). Scalable molecular dynamics with NAMD. J. Comput. Chem. 26, 1781–1802. doi: 10.1002/jcc.20289 16222654 PMC2486339

[B31] Phillips J. C. Hardy D. J. Maia J. D. C. Stone J. E. Ribeiro J. V. Bernardi R. C.. (2020). Scalable molecular dynamics on CPU and GPU architectures with NAMD. J. Chem. Phys. 153, 044130. doi: 10.1063/5.0014475 32752662 PMC7395834

[B32] Plotly Technologies Inc (2015). Collaborative data science (Montreal, QC: Plotly Technol Inc.).

[B33] Qi D. de Young B. J. Innes R. W. DeYoung B. J. Innes R. W. (2012). Structure-function analysis of the coiled-coil and leucine-rich repeat domains of the RPS5 disease resistance protein. Plant Physiol. 158, 1819–1832. doi: 10.1104/pp.112.194035 22331412 PMC3320188

[B34] Schultink A. Qi T. Bally J. Staskawicz B. (2019). Using forward genetics in nicotiana benthamiana to uncover the immune signaling pathway mediating recognition of the xanthomonas perforans effector XopJ4. New Phytol. 221, 1001–1009. doi: 10.1111/nph.15411 30156705

[B35] Sillitoe I. Bordin N. Dawson N. Waman V. P. Ashford P. Scholes H. M.. (2021). CATH: increased structural coverage of functional space. Nucleic Acids Res. 49, D266–D273. doi: 10.1093/nar/gkaa1079 33237325 PMC7778904

[B36] Slootweg E. Koropacka K. Roosien J. Dees R. Overmars H. Lankhorst R. K.. (2017). Sequence exchange between homologous NB-LRR genes converts virus resistance into nematode resistance, and vice versa. Plant Physiol. 175, 498–510. doi: 10.1104/pp.17.00485 28747428 PMC5580749

[B37] Slootweg E. J. Spiridon L. N. Martin E. C. Tameling W. I. L. Townsend P. D. Pomp R.. (2018). Distinct roles of non-overlapping surface regions of the coiled-coil domain in the potato immune receptor Rx1. Plant Physiol. 178, 1310–1331. doi: 10.1104/pp.18.00603 30194238 PMC6236623

[B38] Slootweg E. J. Spiridon L. N. Roosien J. Butterbach P. Pomp R. Westerhof L.. (2013). Structural determinants at the interface of the ARC2 and leucine-rich repeat domains control the activation of the plant immune receptors Rx1 and Gpa2. Plant Physiol. 162, 1510–1528. doi: 10.1104/pp.113.218842 23660837 PMC3707565

[B39] Sun Y. Li L. Macho A. P. Han Z. Hu Z. Zipfel C.. (2013). Structural basis for flg22-induced activation of the arabidopsis FLS2-BAK1 immune complex. Science 342, 624–628. doi: 10.1126/science.1243825 24114786

[B40] Takken F. L. W. Tameling W. I. L. (2009). To nibble at plant resistance proteins. Science 324, 744–745. doi: 10.1126/science.1171666 19423813

[B41] Tareen A. Kinney J. B. (2020). Logomaker: beautiful sequence logos in Python. Bioinformatics 36, 2272–2274. doi: 10.1093/bioinformatics/btz921 31821414 PMC7141850

[B42] van der Biezen E. A. Jones J. D. G. (1998). The NB-ARC domain: a novel signalling motif shared by plant resistance gene products and regulators of cell death in animals. Curr. Biol. 8, R226–R228. doi: 10.1016/S0960-9822(98)70145-9 9545207

[B43] van Ooijen G. Mayr G. Kasiem M. M. A. Albrecht M. Cornelissen B. J. C. Takken F. L. W. (2008). Structure–function analysis of the NB-ARC domain of plant disease resistance proteins. J. Exp. Bot. 59, 1383–1397. doi: 10.1093/jxb/ern045 18390848

[B44] Wang W. Chen L. Fengler K. Bolar J. Llaca V. Wang X.. (2021). A giant NLR gene confers broad-spectrum resistance to phytophthora sojae in soybean. Nat. Commun. 12, 6263. doi: 10.1038/s41467-021-26554-8 34741017 PMC8571336

[B45] Wang J. J. Hu M. Wang J. J. Qi J. Han Z. Wang G.. (2019a). Reconstitution and structure of a plant NLR resistosome conferring immunity. Science 364, 1–11. doi: 10.1126/science.aav5870 30948527

[B46] Wang J. Li H. Han Z. Zhang H. Wang T. Lin G.. (2015b). Allosteric receptor activation by the plant peptide hormone phytosulfokine. Nature 525, 265–268. doi: 10.1038/nature14858 26308901

[B47] Wang G. Roux B. Feng F. Guy E. Li L. Li N.. (2015a). The decoy substrate of a pathogen effector and a pseudokinase specify pathogen-induced modified-self recognition and immunity in plants. Cell Host Microbe 18, 285–295. doi: 10.1016/j.chom.2015.08.004 26355215

[B48] Wang J. Wang J. Hu M. Wu S. Qi J. Wang G.. (2019b). Ligand-triggered allosteric ADP release primes a plant NLR complex. Science 364 (6435), eaav5868. doi: 10.1126/science.aav5868 30948526

[B49] Webb B. Sali A. (2016). Comparative protein structure modeling using MODELLER. Curr. Protoc. Bioinforma 54, 5.6.1–5.6.37. doi: 10.1002/cpbi.3 PMC5031415 27322406

[B50] Williams C. J. Headd J. J. Moriarty N. W. Prisant M. G. Videau L. L. Deis L. N.. (2018). MolProbity: More and better reference data for improved all-atom structure validation. Protein Sci. 27, 293–315. doi: 10.1002/pro.3330 29067766 PMC5734394

[B51] Williams S. J. Sornaraj P. DeCourcy-Ireland E. Menz R. I. Kobe B. Ellis J. G.. (2011). An autoactive mutant of the m flax rust resistance protein has a preference for binding ATP, whereas wild-type m protein binds ADP. Mol. Plant-Microbe Interact. 24, 897–906. doi: 10.1094/MPMI-03-11-0052 21539434

[B52] Wróblewski T. Spiridon L. Martin E. C. Petrescu A.-J. J. Cavanaugh K. Truco M. J.. (2018). Genome-wide functional analyses of plant coiled–coil NLR-type pathogen receptors reveal essential roles of their n-terminal domain in oligomerization, networking, and immunity. PloS Biol. 16, e2005821. doi: 10.1371/journal.pbio.2005821 30540748 PMC6312357

[B53] Xu D. Nussinov R. (1998). Favorable domain size in proteins. Fold Des. 3, 11–17. doi: 10.1016/S1359-0278(98)00004-2 9502316

[B54] Zhang Y. Cheng T. C. Huang G. Lu Q. Surleac M. D. Mandell J. D.. (2019). Transposon molecular domestication and the evolution of the RAG recombinase. Nature 569, 79–84. doi: 10.1038/s41586-019-1093-7 30971819 PMC6494689

[B55] Zhao X. Chen Z. Wu Q. Cai Y. Zhang Y. Zhao R.. (2021). The sw-5b NLR nucleotide-binding domain plays a role in oligomerization, and its self-association is important for activation of cell death signaling. J. Exp. Bot. 72, 6581–6595. doi: 10.1093/jxb/erab279 34115862

