# Supplementary material for: Tsw – A case study on structure-function puzzles in plant NLRs with unusually large LRR domains
Source: Front Plant Sci. 2022 Oct 7;13:983693. doi: 10.3389/fpls.2022.983693 (PMC9585916; doi:10.3389/fpls.2022.983693)
Supplement: Supplementary file 1 [file DataSheet_1.pdf]

Supplementary Material

Supplementary Figure S1

A **Taxonomic spread**

Extended Tsw homologs
> 30% identity

Close Tsw homologs
> 50% identity

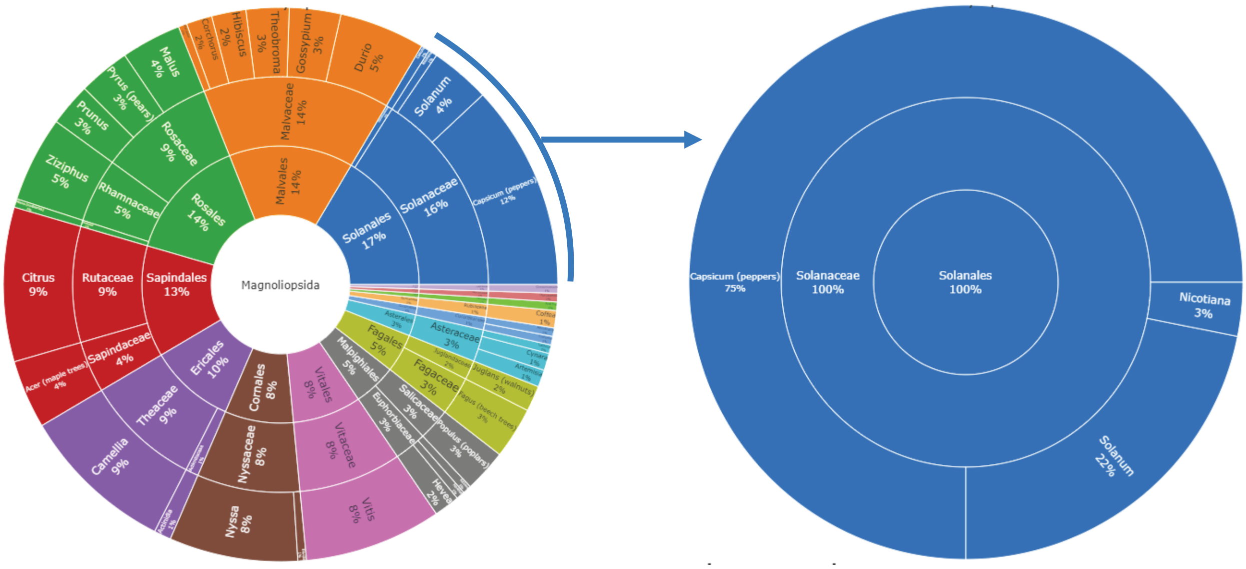

200 nonredundant members
(90% identity)

32 nonredundant members
(90% identity)

B

Sequence variability

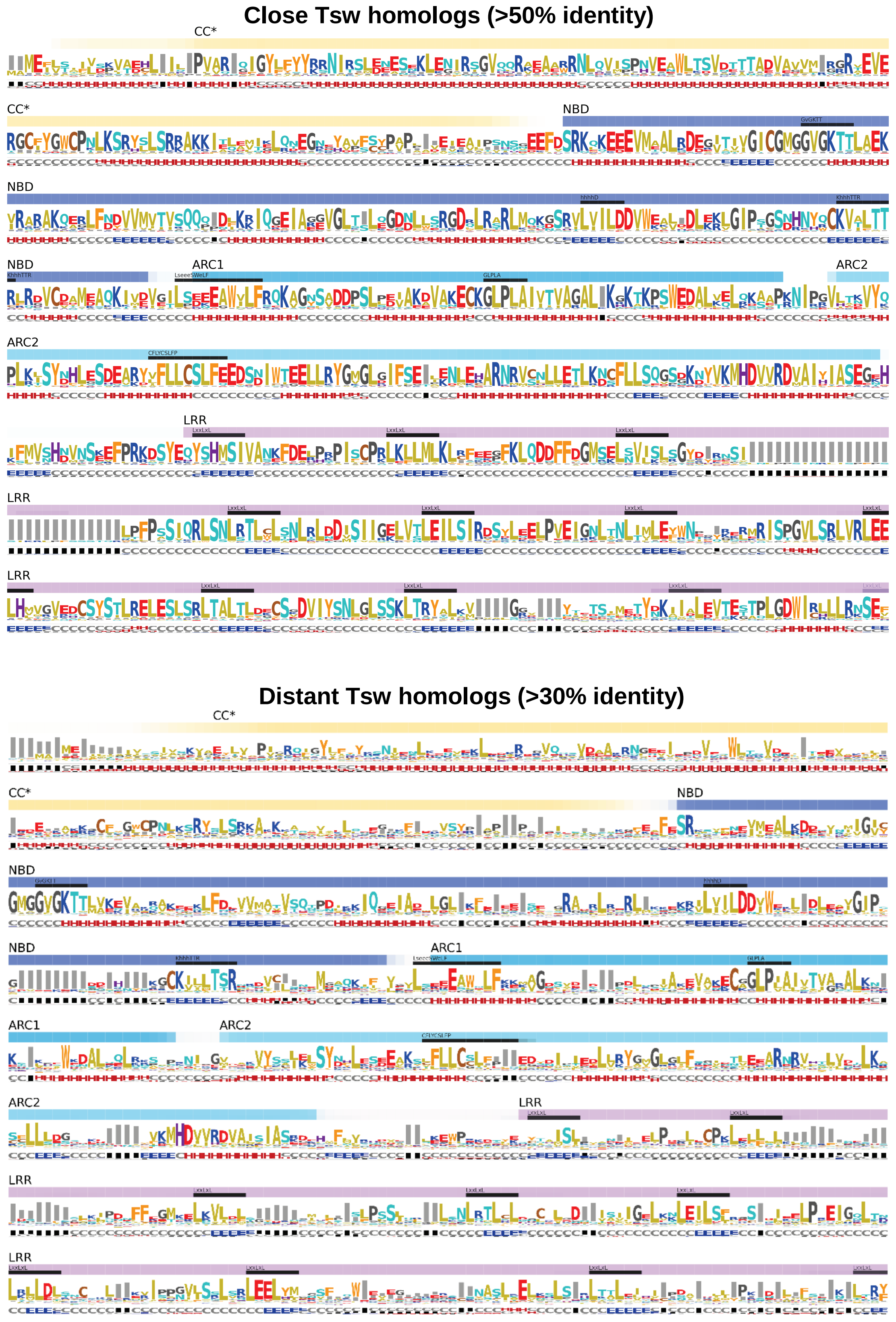

*Displayed are only the first 8 LRR repeats, due to increased gap percentage over the rest of the LRR domain alignment

C **LRR motif variability**

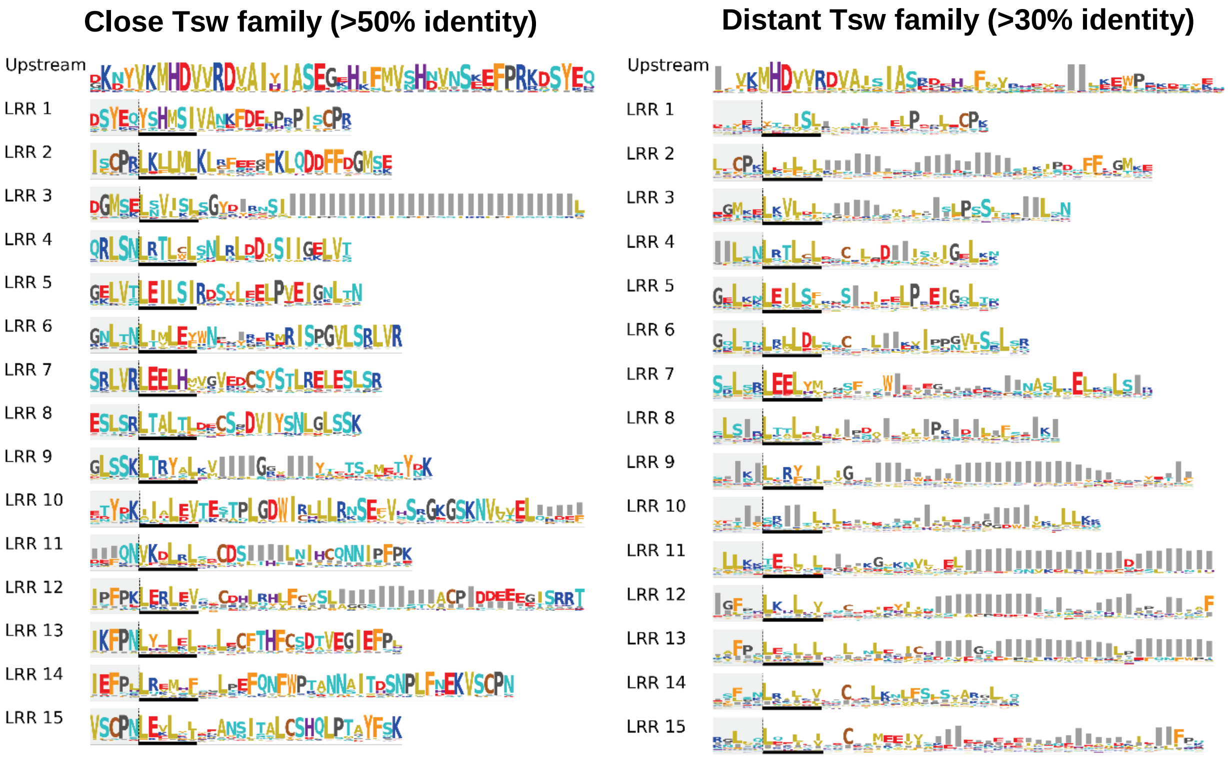

*Displayed are only the first 15 LRR motifs

D Identity matrix – extended set of homologs

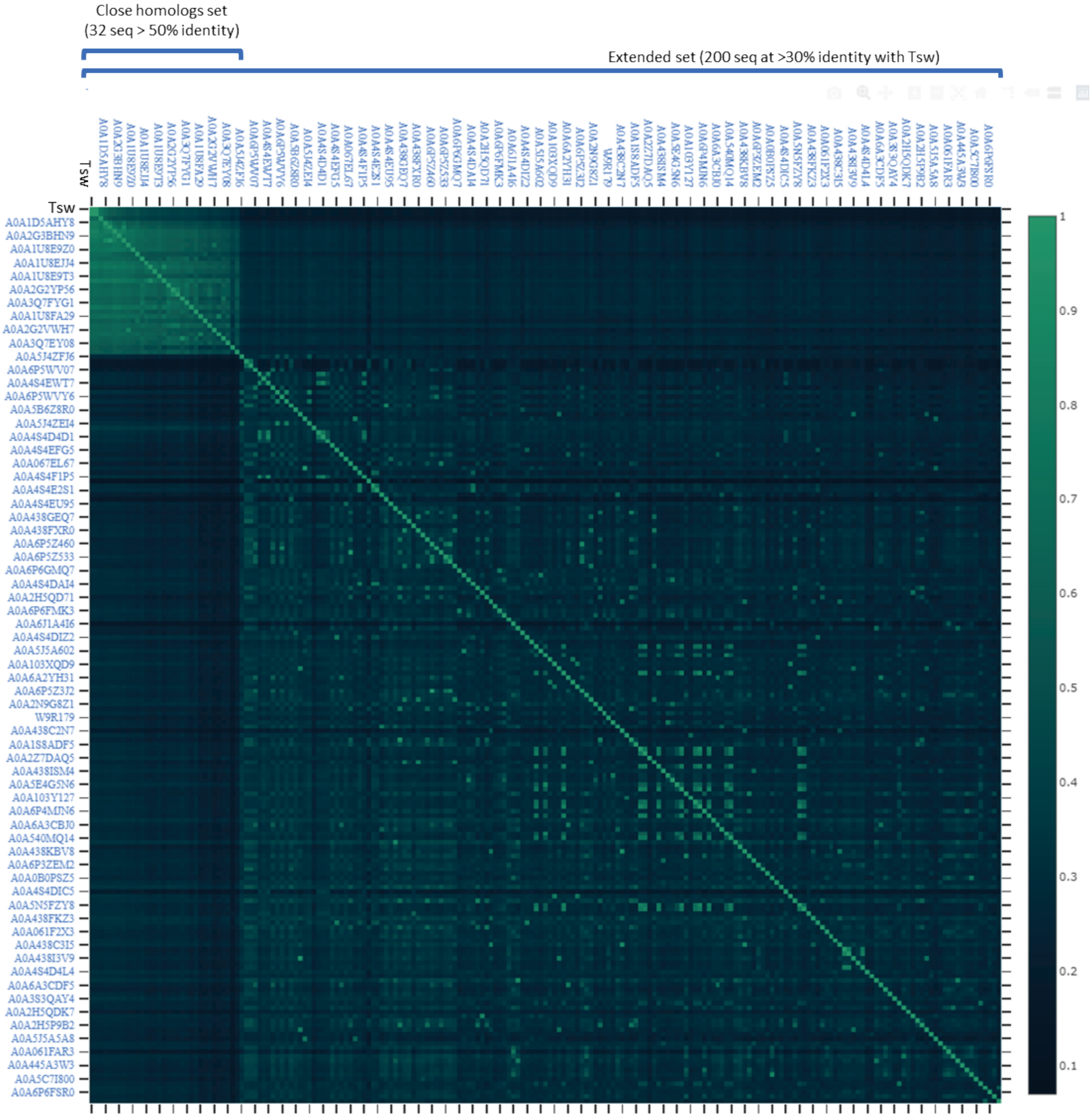

*Only several labels are visible (indicated by tickmarks), due to the large size of the matrix.

** Identity is computed as the ration between the number of identicatl residues between Seq1 and Seq2 over the length of Seq1 (lower diagonal side) or of Seq2 (upper diagonal side). Therefore the matrix is not symmetrical.

Supplementary Figure S1. Tsw variability analysis on two sets of homologs: an extended homologs group of 200 sequences sharing between 30-90% identity with Tsw and subset of it corresponding to 32 close homologs subset which share 50-90% identity with Tsw: (A) Taxonomy distribution, (B) Variability plots, (C) LRR motif variability and (D) Identity matrix.

Supplementary Figure S2

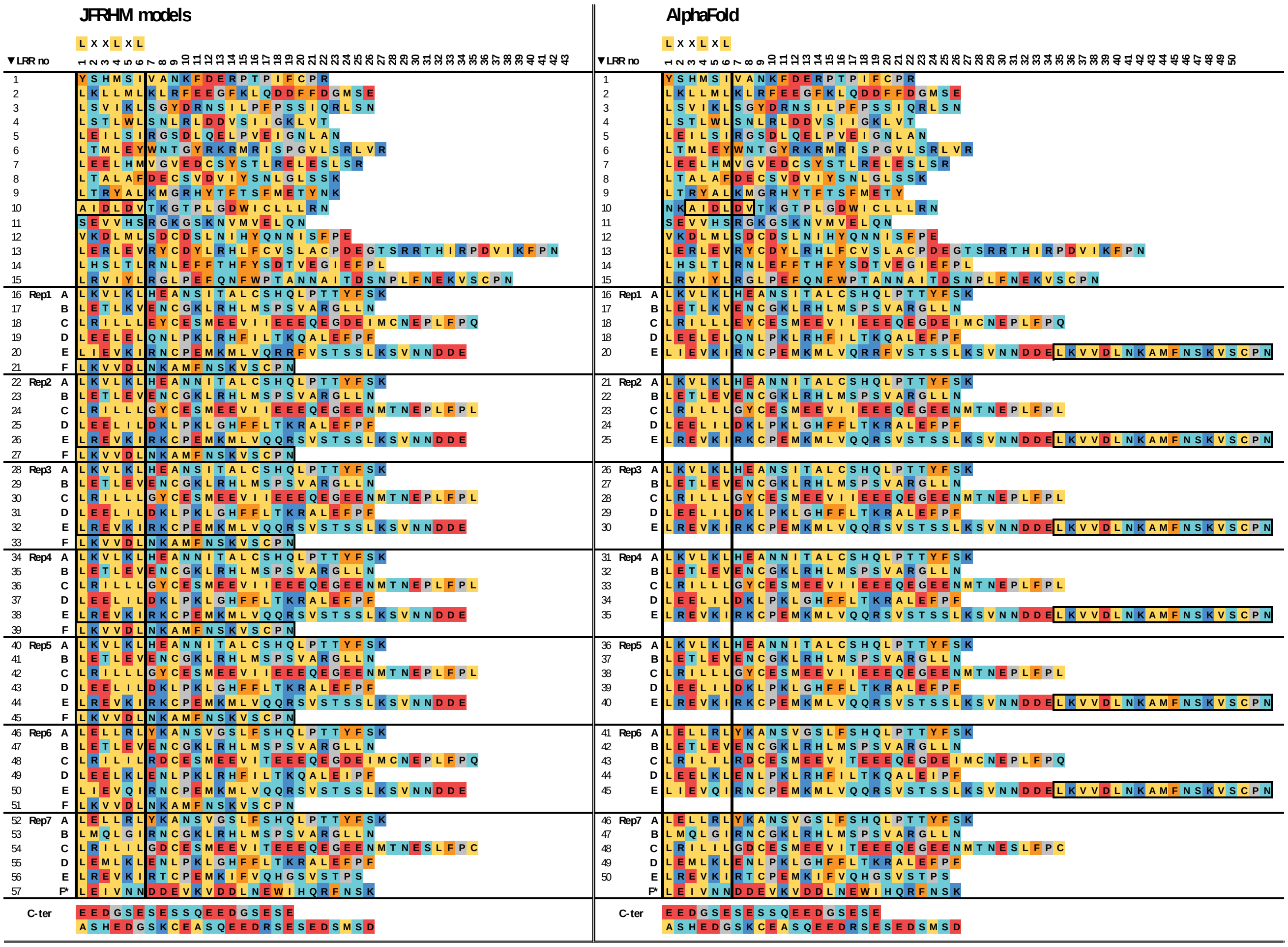

Supplementary Figure S2. The Tsw LRR repeat delineation used by AlphaFold and the adapted JFRHM models. Differences are depicted by black boxes.
